# Reaching out to a community to improve maternal health in Ghana: the story of one midwife

**DOI:** 10.9745/GHSP-D-14-00110

**Published:** 2014-08-31

**Authors:** John Kuumuori Ganle

**Affiliations:** a University of Oxford, Nuffield Department of Population Health, The Ethox Centre, Oxford, UK

The story below of a Ghanaian midwife from the Ashanti region illustrates how one person was able to mobilize local community members in rural Piase in the Bosomtwi district to create demand for, and improve access to and use of, emergency and routine maternal health services. Her story demonstrates how involving communities in maternal health issues can improve both access to services and maternal health outcomes.

“When I was first posted to Piase in 2005 as a midwife, attendance at antenatal care (ANC) clinics was very low. Women were also delivering their babies at home. So I became very worried. I was particularly worried because maternal and newborn care services were provided free-of-charge at the health facility. I therefore started asking a lot of questions regarding why the women were not using our services.

I discovered that the women were not coming because of certain things that were happening in the community. For example, I found out that they [the women] were using the services of TBAs [traditional birth attendants], and these TBAs were actively discouraging the women from coming to the health facility to receive skilled care. The surprising thing I also found was that some religious leaders, I mean pastors, were discouraging their congregants from using health facility services [and instead encouraging them to find relief] through prayers and spiritual healing.

I was indeed very worried, and this was so because the midwife who came to the community before I did had a very bad relationship with the TBAs, religious leaders, and some traditional or opinion leaders in the community. The stories I have heard are that the community members complained that my predecessor showed gross disrespect for the culture and traditional authority structures of the community. In addition, I was told the midwife had several confrontations with the TBAs, with most of them complaining that the midwife had come to take away their jobs and source of livelihood.

As a form of protest and discontent, TBAs, pastors, and traditional authorities, particularly the queen mother, embarked on a silent campaign that sought to discourage pregnant women from accessing and using the services that were being provided at the health facility. When I discovered all these, I didn't really know what to do to make things better … In fact, I was scared, and I wanted to seek transfer to a different community.

But after thinking through the problem, I decided that I would stay in the community and reach out to the traditional leaders, TBAs, pastors, and women themselves. So I visited the chief, queen mother, assemblymen, and each of the TBAs and pastors in the community to first introduce myself to them as the new midwife. I also visited women's groups in the community and churches to introduce myself. During these introductory meetings, I asked them about what they thought the problems of maternal health care were and how we could come together to work to make things better. You know, the TBAs were very surprised that I was asking for their opinions because I was the midwife and I was supposed to know everything. But I said that, ‘Well, I might not know everything,’ and given that I am new in the community, I believe they [the TBAs] could be of immense help. I did the same thing with the pastors. In fact, I organized a meeting and invited all the pastors, TBAs, community leaders, and women to discuss how best we could ensure that no woman suffers or dies as a result of pregnancy and childbirth.

During the meeting, I made it clear that they should see the problem of maternal health as belonging to all of us, and that they too could help. Because of the problems my predecessor had with the TBAs and also because the Ghana Health Service in the district does not recognize TBAs as skilled caregivers, I reassured all the TBAs that I did not come into their community to take their jobs but to work with them, so that together we could make things better. For the pastors too, I told them that their prayers too were still important during pregnancy or labor. I told them that I was ready to assist with the physio-biological aspects of the health of mother and newborn while they [the pastors] deal with spiritual matters.

After these individual meetings, I sought permission from the chief and queen mother to organize a *durbar*. This was a big ceremonial meeting, which was attended by several of the community members, especially husbands and wives, and was marked with celebrations, food, and traditional dances. During this meeting, we held discussions on the importance of women accessing and using skilled care services during pregnancy, delivery, and thereafter, the barriers to services use, and how they [the community members] could act as partners with us, the health care providers, to improve both access to care and birth outcomes.

Following this big meeting, we formed a community maternal health watchdog committee. The chairperson for the committee was the queen mother, and the committee was tasked to undertake house-to-house sensitization and home visits to identify pregnant women and encourage them to attend ANC clinics. The committee also monitored and reported on issues related to maternal and newborn health such as miscarriage and abortion. On my part, I worked closely with the committee, pastors, and TBAs.

At the beginning, many of the community leaders and TBAs were very reluctant to engage with me on the issue of maternal health. But I continued to consult with them as well as to express my desire for us to work together as partners. I even went to help some of the TBAs conduct deliveries at home. It was through this that the TBAs came to realize that I had some skills that they did not have. So gradually, the TBAs and pastors started to encourage pregnant women to come for ANC … Now the TBAs themselves will even bring the women to the clinic to deliver. I am really surprised. And the same thing is happening with the pastors. They are also encouraging the pregnant women in their various churches to attend ANC. When a woman goes into labor too, they usually come along to pray for a smooth delivery. So now, we are all working like a team, and I can say that it has contributed a lot to all the progress we are making in this community.

In addition to the fact that the community members and myself have become partners in promoting access to and improving maternal health, no maternal or neonatal death has occurred in the community since 2007. I believe we have achieved this success because of the increase in the proportion of women who now deliver at health facilities.

**Figure:**
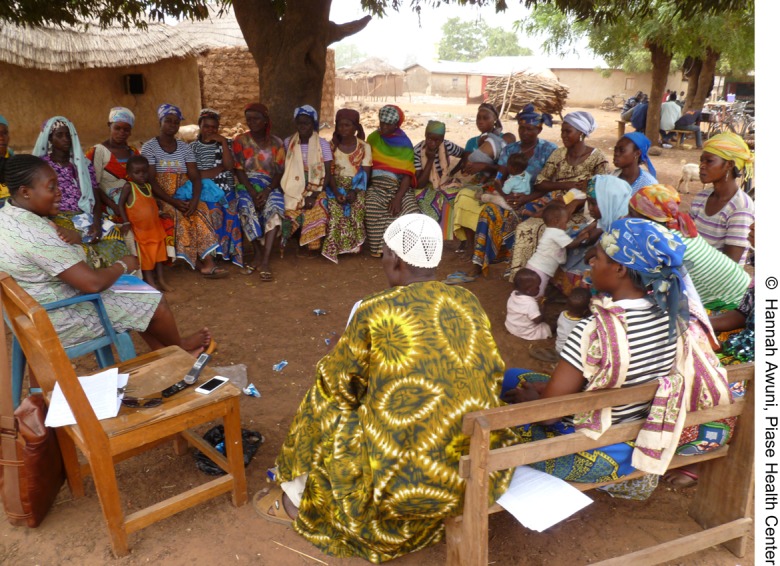
A midwife engages the community chief, queen mother, and women from the community on how to improve maternal health.

**Figure:**
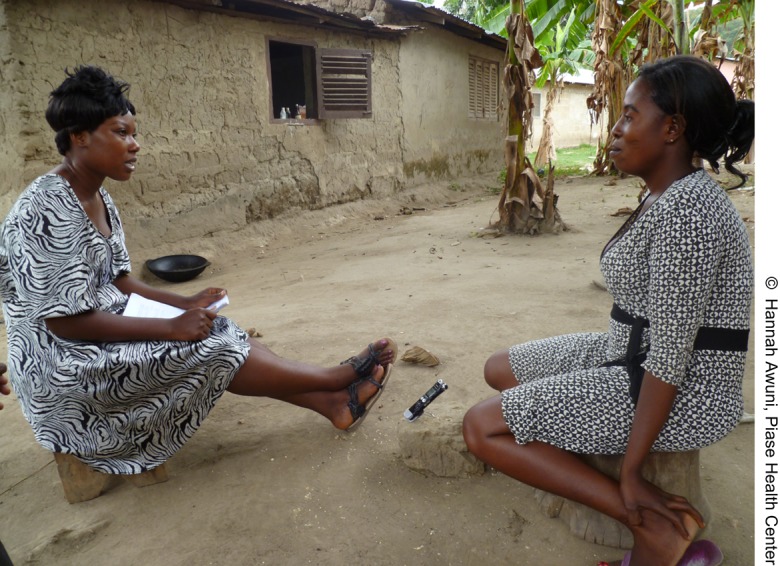
A midwife consults with a community leader on how to encourage women to seek skilled maternal health care services.

Of course I know that my approach is slightly alien to the health system in Ghana and might therefore not be accepted in some contexts. But the modest gains we have attained in this community suggests the need for the Ghana Health Service and us, the individual health care providers, to do more to build partnerships with traditional midwives or TBAs, traditional and religious leaders, as well as community members on the issues of maternal health. For me, partnerships means shared responsibility between us, the health care providers, and individual women and communities, and this can offer us opportunities to change women's as well as local communities' beliefs and attitudes toward hospital-based maternity care services. My own experience over the years as a woman who has gone through pregnancy and childbirth and who has worked at both urban and rural settings as a frontline midwife providing maternity care services has taught me that community engagement and partnership is the only way to enhance the potential for cooperation between health care workers and community members, as well as to increase opportunities for nurses and midwives to train TBAs to increase their skills to offer competent and safe maternity care services, including responsive referrals of cases at the community level. My personal belief is that if we want to make progress with maternal health, then we must begin to foster more collaboration between nurses, midwives, TBAs, and traditional and religious leaders at the community level.”

## KEY PROGRAMMATIC LESSONS

The story of this midwife is drawn from my 6-month research journey starting in November 2011, from my English town of Oxford to the West African country of Ghana. My primary goal was to elicit and document the experiences and views of a wide range of people on why the maternal mortality rate was still very high, and accessibility to and use of skilled maternal health care services was low despite the implementation of a free maternal health care policy in Ghana since 2003.^1^^–^^3^ I conducted focus group discussions, interviews, and structured field observations with 185 expectant and lactating mothers, 15 TBAs, 12 traditional and opinion leaders, and 20 health care providers (i.e., community health nurses, midwives, doctors, health facility managers, district and regional directors of health, district and regional public health nurses, and policy makers at the Ministry of Health and Ghana Health Services).

The approach adopted by the midwife highlighted in this story did not merely raise community awareness or persuade community members to participate in activities that the midwife and the local health delivery system had already designed or decided on. Rather, her approach involved a comprehensive consultative and participatory strategy that involved a series of activities including:

1. Conducting formative research to understand factors within the community that constrain women's access to and use of services
2. Entering the community and establishing credibility and trust
3. Galvanizing the support of traditional authorities in the community mobilization process
4. Working with community leaders and other stakeholders to invite childbearing women and organize their participation in strategic activities
5. Monitoring and evaluation of community maternity care activities

The processes the midwife followed showed great respect for community members and acknowledged and/or used existing local structures, resources, and networks to generate interest in, and demand for, skilled maternal health care services. Although there could be challenges such as lengthy processes and community members' unwillingness to engage in the process, this particular midwife's story suggests that the overall potential benefits of engaging with target communities outweigh the challenges.

As efforts to improve maternal health continue, it is essential that local communities are engaged to generate support and demand for skilled birthing services as well as create the needed behavioral and attitudinal changes.
